# A Strategic Approach to Addressing Aggressive Vertebral Hemangiomas With Hypofractionated Stereotactic Body Radiotherapy

**DOI:** 10.7759/cureus.56877

**Published:** 2024-03-25

**Authors:** Hadia Fatima, Abdulrahman Alhadab, Salem M Alshehri

**Affiliations:** 1 Radiation Oncology Department, King Abdulaziz Medical City, Ministry of National Guard Health Affairs, Riyadh, SAU

**Keywords:** stereotactic ablative radiotherapy fractionation, hypofractionation, vertebral hemangioma, cyberknife stereotactic hypofractionated radiotherapy, spinal neoplasms, aggressive vertebral hemangioma

## Abstract

This case report describes the treatment of a recurrent T2 vertebral hemangioma in a 46-year-old man who had prior decompression and fusion surgery. Despite initial stability, the patient developed worsening symptoms, leading to a comprehensive approach involving embolization, microscopic excision, and posterior fixation. Recurrence prompted the choice of Stereotactic Body Radiotherapy (SBRT) over redo surgery. Administered with 30 Gy in five fractions, SBRT significantly reduced hemangioma size and resolved neurological symptoms. The case highlights the effectiveness of hypofractionated SBRT as a promising intervention for aggressive vertebral hemangiomas.

## Introduction

Vertebral hemangiomas are common benign vascular tumors often discovered incidentally, with an estimated incidence of 1.9%-27% [1]. While most are asymptomatic or present with isolated pain, less than 1% become aggressive, termed atypical vertebral hemangiomas (AVH), potentially compressing the spinal cord. AVH is classified under Enneking 3, S3 [1]. Recent multicenter studies report a low local recurrence rate of 2.9%, contrasting earlier findings suggesting rates up to 27.3% [2]. Stereotactic Body Radiotherapy (SBRT) has emerged as a valuable intervention for aggressive cases. Despite its potential, literature on hypofractionated SBRT for vertebral hemangiomas is limited, with only 24 reported cases to date [3]. This case report aims to contribute to this scarce body of knowledge, underscoring the significance of hypofractionated SBRT in managing aggressive vertebral hemangiomas.

## Case presentation

**Figure 1:**
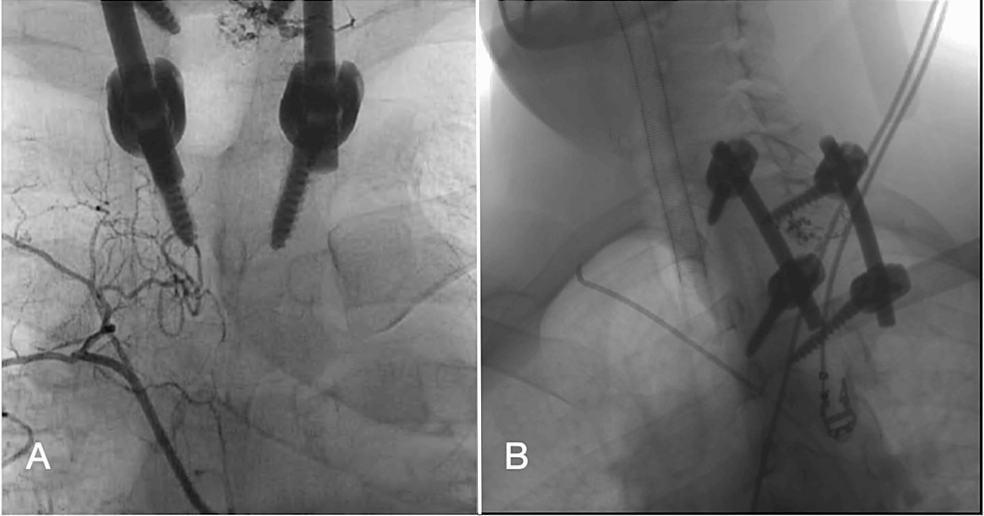
Pre (A) and post (B) embolization of three small feeding arterial branches to T2 vertebra hemangioma using coils led to approximately 60% reduction of the pathological blood supply.

A 46-year-old male, with no known comorbidities underwent decompression and fusion surgery for a thoracic spine hemangioma 1.5 years ago, presented to the emergency department with a two-week history of progressive backache (pain Score 8/10), lower limb pain, urinary hesitancy, and an unsteady gait. Initial Imaging with Thoracic Spine MRI showed post-surgery, stable changes, but a T2 vertebral body lesion persisted, causing significant spinal canal narrowing, bilateral neural foramina compression, and spinal cord compression. A subsequent Thoracic Angiogram revealed a lesion enhancement at the T2 vertebral body level, supplied by small branches from the right costocervical artery and the right spinal branch of T3. Additionally, the radicular artery was connected to the anterior spinal artery at the left soil branch of T3. There was a 60% reduction in blood supply following pre-operative embolization (Figures 1A, 1B).

**Figure 2:**
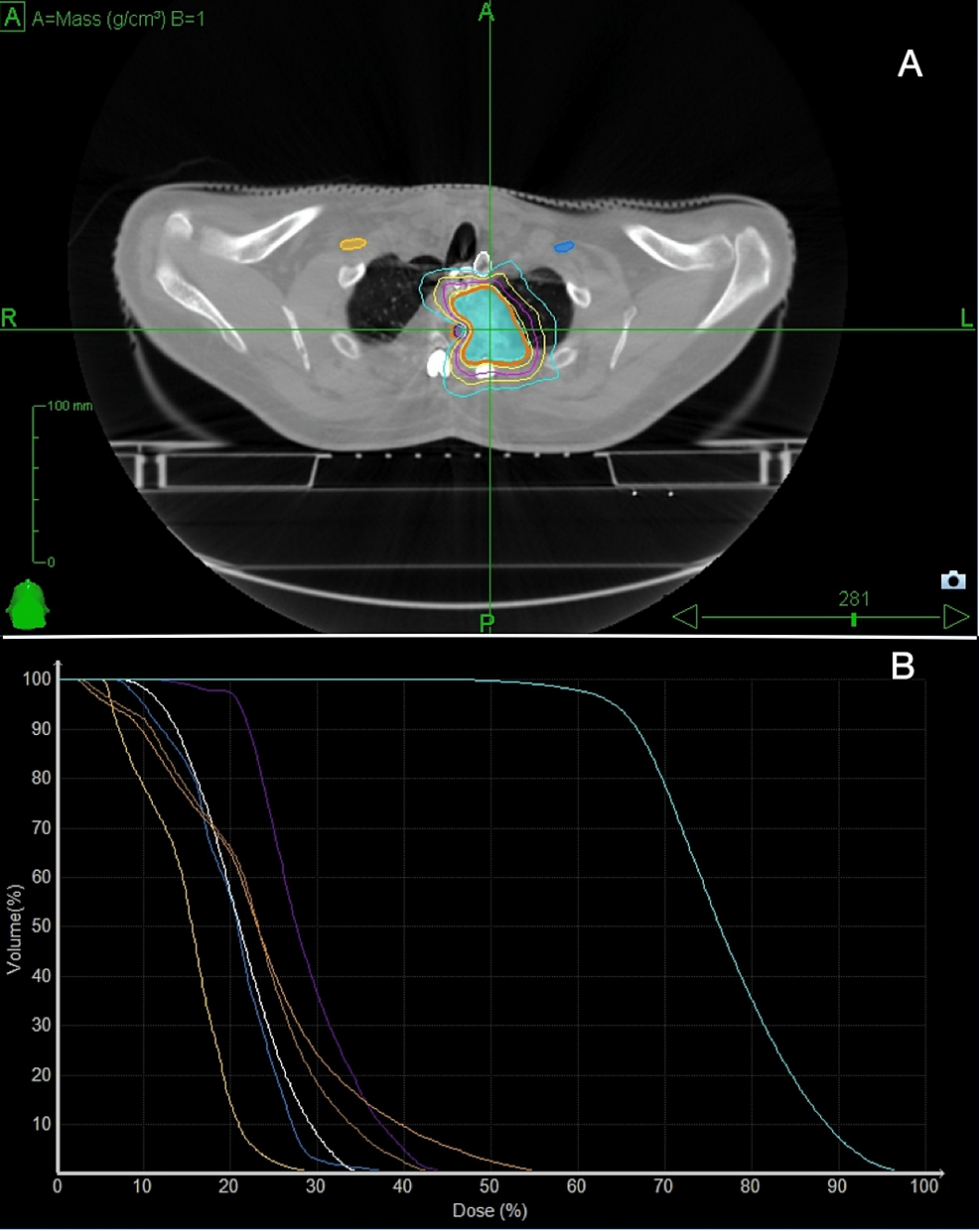
Dose color wash (A) and DVH (B) images from hypofractionated SBRT planning. Colors of Isodose Lines Red: GTV; Light Blue: PTV;  Navy Blue: Left Brachial Plexus; Mustard: Right Brachial Plexus; Purple: Spinal Cord; Orange: PRV Cord; Brown: Cord plus 5 mm; Esophagus: White

Microscopic excision, decompression, T1-T3 posterior fixation, and T2 vertebral body corpectomy were performed. The patient had an uncomplicated postoperative course, initiating ambulation with physiotherapy. Postoperative follow-up MRI revealed Partial resection of the T2 vertebral body hemangioma with instrumentation, which showed interval improvement in edematous changes. The spinal canal at the surgical level appeared more capacious. The patient was kept on active surveillance due to no remnant disease post-surgery. Serial MRI showed residual osseous hemangioma recurrence after 10 months, with mild enlargement involving the left pedicle, lamina, and left aspect of T2 vertebral body. The patient presented with moderately severe pain extending towards the back, rated at 6/10, without any accompanying neurological symptoms. Given recurrence, the multidisciplinary tumor board opted for Stereotactic Body Radiotherapy (SBRT) instead of redo surgery. SBRT to T2 lesion with 30 Gy in five fractions was completed (Figures 2A, 2B, Table 1).

**Table 1:** Dose statistics

Structure	Min (cGy)	Mean (cGy)	Max (cGy)	CI	HI	Coverage%
GTV	2,426	3,656	4,545	1.44	1.52	98.64
PTV	1,480	3,493	4,545	1.08	1.52	91.85
Rt. Brachial Plexus	282	920	1,827	-	-	-
Lt. Brachial Plexus	205	677	1,404	-	-	-
Spinal Cord	493	1,300	2,107	-	-	-
PRV Cord	107	1,075	2,684	-	-	-
Esophagus	334	964	1,613	-	-	-

**Figure 3:**
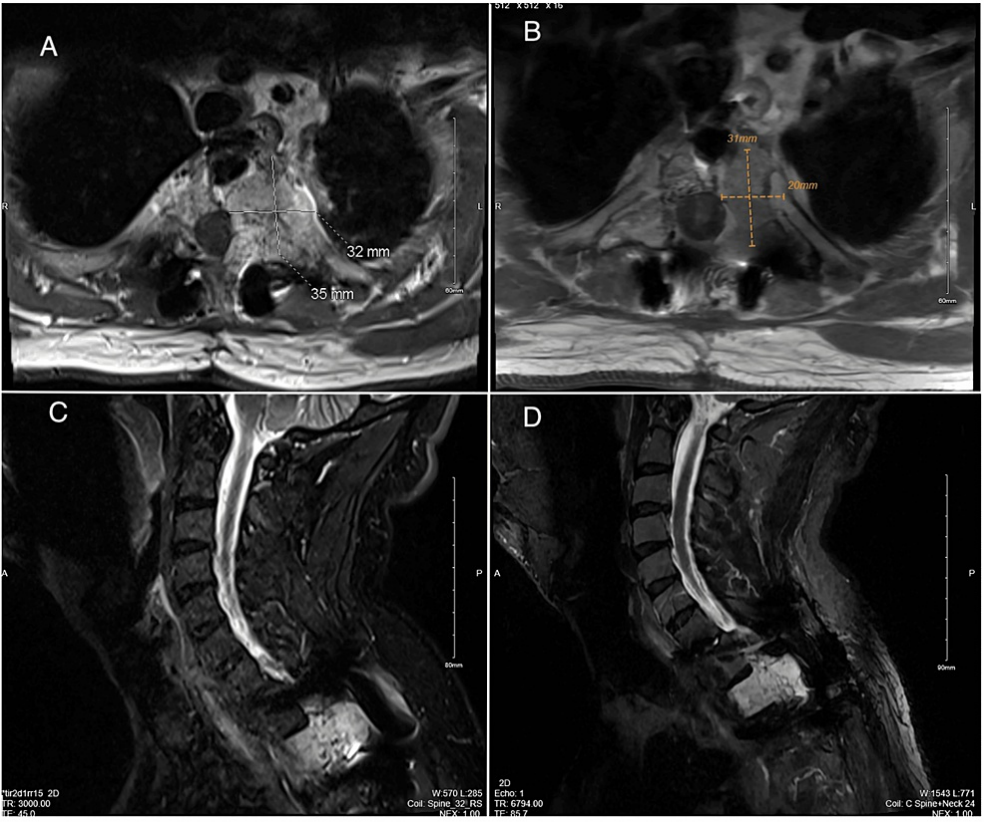
Post contrast T1 weighted pre (A, C) and post (B, D) hypofractionated SBRT MRI images of thoracic spine showing interval disease regression.

After six months, an MRI for response evaluation showed mature postoperative changes. The T2 hemangioma showed a mild interval decrease in size with reduced epidural extension, correlating with clinical improvement. The patient tolerated and responded well to SBRT, experiencing a decrease in the size of the hemangioma and remarkable improvement in symptoms. Pain score significantly improved from 6/10 to 1/10 with no observed neurological deficit or deformity (Figures 3A-3D).

## Discussion

Vertebral hemangiomas are noncancerous vascular lesions originating from abnormal embryonic development and are commonly found in the thoracic and lumbar spine. In radiological studies, they are discovered incidentally, in approximately 11% of autopsy cases. While the majority are asymptomatic, about 0.9% to 1.2% may cause symptoms, typically presenting as back pain or neurological issues [4]. Histologically categorized as cavernous or capillary angiomas, these lesions exhibit a palisading appearance on X-rays, a “honeycomb” or “polka dot” pattern on CT scans, and high signal intensity on both T1- and T2-weighted MRI sequences. Encased by a capsule, they involve thick-walled vessels infiltrating the marrow [4].

Symptomatic vertebral hemangiomas can be classified into three types: latent (mild bony destruction without symptoms), active (bony destruction with pain), and aggressive (neurological deficit with epidural and/or soft tissue involvement). Acute or subacute neurological symptoms are rare, occurring in less than 1% of cases. In clinical practice, Enneking staging (SI, SII, SIII) is often used to categorize the lesions based on their severity and symptomatology [5]. This classification helps guide appropriate management strategies for patients with vertebral hemangiomas. Symptoms manifest in fewer than 2% of cases, prompting the need for intervention. Surgical procedures, radiation therapy, radiofrequency ablation, intra-lesional ethanol injection, or a combination of these therapies may be considered (Table 2) [6].

**Table 2:** Management options for vertebral hemangiomas Credits: Hadia Fatima

Treatment Modality	Advantages	Limitations	Patient Suitability
Active Surveillance	Avoids intervention related risks	Monitoring required	Asymptomatic, Small Lesions
Embolization	Minimally Invasive, Good Vascular Control, Preoperative embolization is done to shrink the tumor	Limited applicability to certain cases, Recurrence when used as a single modality treatment, Stroke, Peripheral arterial occlusion, Cord ischemia and Allergic reactions to the agents	Significantly Vascular Lesions
Radiofrequency Ablation	Determination of the geometry of the ablation zone beyond which tissues are safe from thermal injury. Mainly used for Osteolytic bone lesions due to lower intrinsic impedance	Transient Post procedure pain, Ablation injuries, Ablation induced fractures	Vertebral lesions with no or small extraosseous components
Intralesional Ethnol	Causes intravascular thrombosis leads to lesion shrinkage, Relieving neurological signs and symptoms, Injection can be repeated again	Rare chance of neurological complication, seizure-like episodes	Significantly Vascular Lesions
Surgery	Deteriorating neurological condition, Spinal canal stenosis, Spinal instability, Prevention of epidural bleeding in patients with a compression fracture of the vertebral body, Neurologically stable cases, Patients with large vertebral body distortions caused by the tumor	Wound healing Complications, Recurrent Myelopathy, Embolism, Hemorrhage, Hypovolemic Shock	Large and Symptomatic Lesions
Radiotherapy	Non-invasive, Precise Targeting As definitive therapy in patients with neurological deficits, Adjuvant treatment in cases of partial Resection	Limited long term follow up data, Slow Neurological Recovery and Overall Response	Small to moderate size lesions

The effectiveness of radiation therapy in addressing symptomatic vertebral hemangiomas stems from its recognized anti-inflammatory impact on these lesions. Prevalent in 86% of cases, hemangiomas often involve more than one-third of the vertebral body. Treatment typically entails 2 Gy daily fractions over four weeks, totaling 40 Gy. A follow-up, spanning a median period of 18 months, revealed substantial improvement in 24 out of 28 patients. Notably, 54% achieved complete pain relief, with an additional 32% exhibiting partial response. No severe acute or late treatment-related side effects were reported [7].

A common practice involves subtotal resection followed by adjuvant radiation therapy to mitigate recurrence rates, which can reach 30%-50% without supplemental radiotherapy [7]. Achieving an equivalent dose in 2 Gy fractions (EQD2) of 40 Gy has been underscored for optimal outcomes, offering symptomatic relief and disease control. Due to its slow growth and benign nature, the alpha/beta ratio of 3 for vascular hemangioma was proposed for utilization in the Linear-Quadratic model [8].

Current radiation therapy techniques, including Image-Guided Radiation Therapy (IGRT), Intensity-Modulated Radiation Therapy (IMRT), and Volumetric Arc Modulated Radiation Therapy (VMAT), are employed for vertebral hemangiomas. These advanced methods, coupled with daily imaging, facilitate the delivery of conventional dose fractionation. Radiosurgery, particularly utilizing CyberKnife, stands out as a safe and effective treatment for symptomatic vertebral hemangiomas. A systematic review and meta-analysis covering studies from 1990 to 2020 highlighted favorable rates of tumor control (94.1%) and pain relief (87.5%) post-radiation therapy. This study suggested that lower radiation doses might lead to pain relapse. One patient experienced symptom relapse following SBRT and received 13 Gy in 1 fraction, while others received higher doses of 18 or 20 Gy in one fraction. Common hypofractionated stereotactic radiotherapy dose regimens include 32 Gy in four fractions or 30 Gy in five fractions. Minimal damage to surrounding tissues was reported (Table 3) [3].

**Table 3:** Clinical outcomes of SBRT in the management of vertebral hemangiomas Credits: Hadia Fatima

Parameters	Response Rates
Local Control: Complete Response	45.7%
Local Control: Partial Response	23.6%
Local Control: Stable Disease	37.2%
Local Control: Progressive Disease	0%
Overall Local Control	94.1%
Pain Control	87.5%
Reossification	27.3%
Damage to surrounding structures	22.3%

Single-fraction SBRT, employing a median dose of 18 Gy, emerges as a viable option for addressing symptomatic vertebral hemangiomas. Patients in this series reported significant symptom improvement, and importantly, no acute or chronic toxicities were associated with this approach [9]. Current clinical trials, such as the one registered on ClinicalTrials.gov (NCT02332408), aim to compare the analgesic effect, toxicity, and pathological impact of conventional radiotherapy schedules (2 Gy per fraction, total dose 36 Gy) and hypofractionated (5 Gy per fraction, total dose 25 Gy) radiotherapy modalities for painful vertebral hemangiomas. The results of this trial will provide insights into optimizing radiotherapy approaches for enhanced patient outcomes [10]. However, the results are pending, and therefore, it is premature to establish standard practices based on its findings. Based on our institutional practice, employing a 30 Gy course administered in five fractions for larger benign spinal tumors has consistently yielded outstanding outcomes, ensuring a fracture risk of no more than 10% [11]. A prolonged observation of patients who underwent SBRT for benign spinal lesions indicated that there were no notable distinctions in local control, pain-flare incidence, or long-term toxicity between low-dose (BED10Gy ≤ 30) and high-dose SBRT treatments [12].

## Conclusions

In addressing the recurrent intraosseous vertebral hemangioma, the decision to opt for SBRT proved successful, aligning with the promising role of hypofractionated SBRT in managing aggressive vertebral hemangiomas. The positive outcomes observed in this case, including decreased hemangioma size and improved neurological symptoms, contribute to the growing evidence supporting the efficacy of hypofractionated SBRT as a valuable alternative to traditional approaches for aggressive vertebral hemangiomas.
